# Rare Complication of a Subcapsular Hepatic Biloma Following Endoscopic Retrograde Cholangiopancreatography for Choledocholithiasis

**DOI:** 10.7759/cureus.86181

**Published:** 2025-06-17

**Authors:** Thoufeer Ali, Mohammed Iqbal, Hameed Rizwan, Imalda Sebastian

**Affiliations:** 1 Internal Medicine, Wrexham Maelor Hospital, Wrexham, GBR; 2 Gastroenterology, Wrexham Maelor Hospital, Wrexham, GBR

**Keywords:** biliary tract disease, endoscopic retrograde cholangiopancreatography (ercp), gastroenterology and endoscopy, post-endocscopy complications, spontaneous biloma

## Abstract

A subcapsular biloma is an uncommon but potentially serious complication of endoscopic retrograde cholangiopancreatography (ERCP). We present the case of a middle-aged woman who developed a symptomatic subcapsular hepatic biloma following ERCP for choledocholithiasis. She was treated with a combination of interventional radiology-guided drainage and repeat ERCP. Early imaging and close clinical monitoring were important to her recovery. This case underscores the importance of maintaining a significant suspicion for atypical post-ERCP presentations and intervening promptly to avoid complications.

## Introduction

Endoscopic retrograde cholangiopancreatography (ERCP) stands as a standard diagnostic and therapeutic procedure for biliary tract diseases like choledocholithiasis. Although pancreatitis, bleeding, and perforation represent typical complications, subcapsular biloma is an exceptionally uncommon event. A biloma represents a bile accumulation that has formed outside the biliary system because of either traumatic or iatrogenic damage [1]. Subcapsular hepatic bilomas that develop without obvious perforation or perihepatic leakage demand cautious diagnostic and therapeutic approaches. This case report analyzes a rare post-ERCP subcapsular hepatic biloma while stressing the importance of diligent post-procedural monitoring and the benefits of interventional radiology and repeat ERCP treatment.

## Case presentation

**Figure 1:**
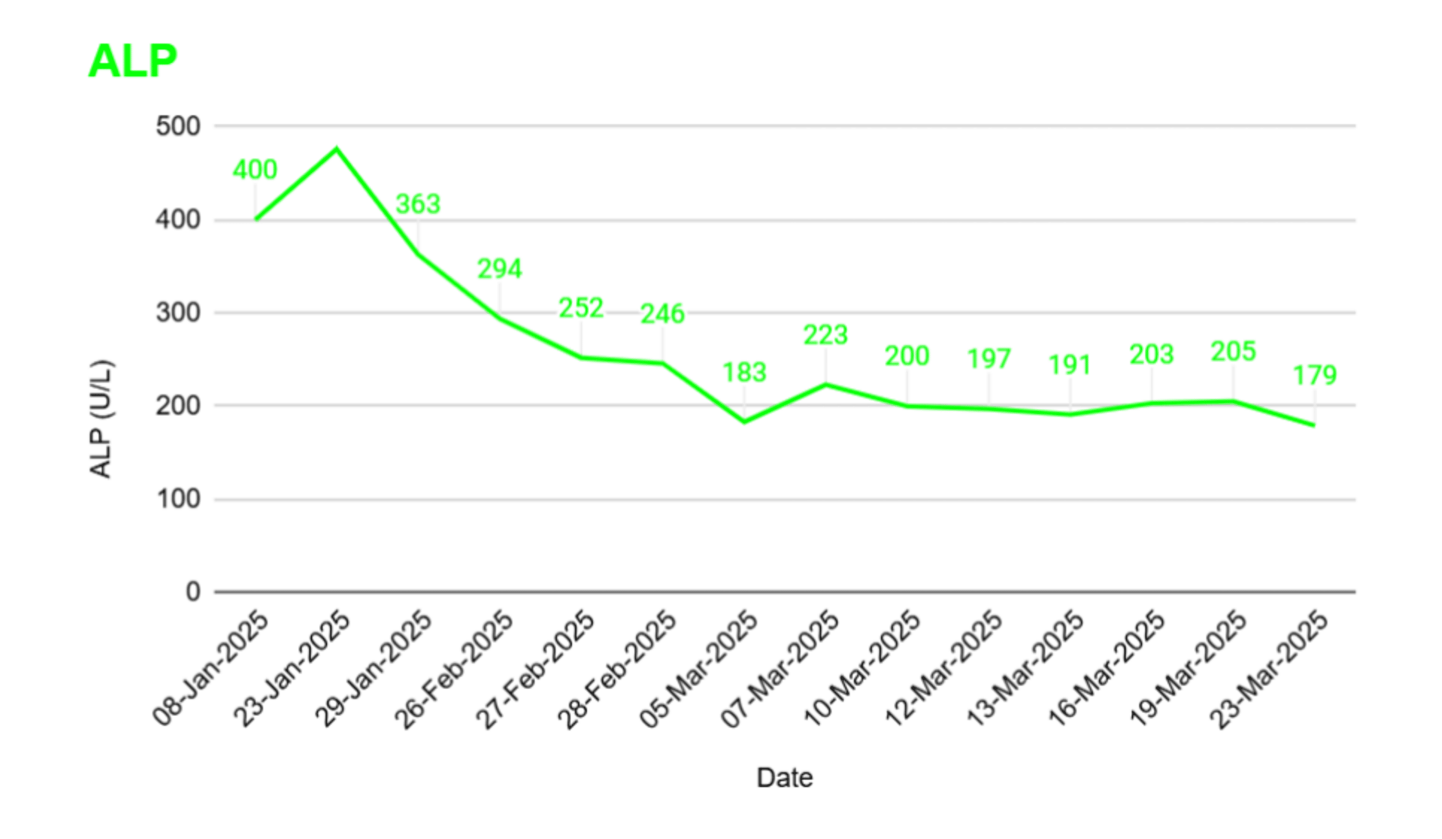
Alkaline phosphatase (ALP) trend over time

A female patient in her early 50s with a history of hiatus hernia, fibromyalgia, gastroesophageal reflux disease, and gallstones presented to the emergency department with a history of continuous retrosternal and right-upper-quadrant pain for the past two days. The initial tests reported elevated levels of liver enzymes, including alanine aminotransferase (ALT), alkaline phosphatase (ALP), and bilirubin, leading to a provisional diagnosis of biliary colic (Figure 1). She was discharged with oral co-trimoxazole (960 mg every 12 hours) and metronidazole (400 mg every eight hours) and planned for outpatient magnetic resonance cholangiopancreatography (MRCP).

The MRCP scan revealed distal common bile duct (CBD) dilation up to 10 mm with multiple distal CBD stones but no intrahepatic dilation. In addition, multiple stones were noted in a non-inflamed gallbladder, so the patient was subsequently referred for ERCP.

Three weeks later, ERCP was performed, successfully achieving selective CBD cannulation. Cholangiogram confirmed multiple distal CBD stones with mild dilation. A sphincterotomy was performed, followed by balloon trawl extraction of an 11.5 mm stone. Multiple stones and a significant amount of sludge were retrieved. Duct clearance was confirmed through occlusive cholangiography.

**Figure 2:**
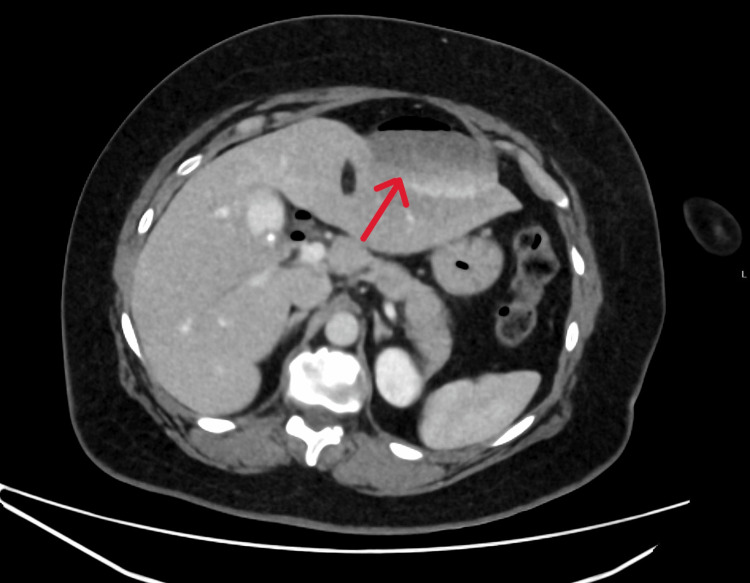
CT image showing a biloma

**Figure 3:**
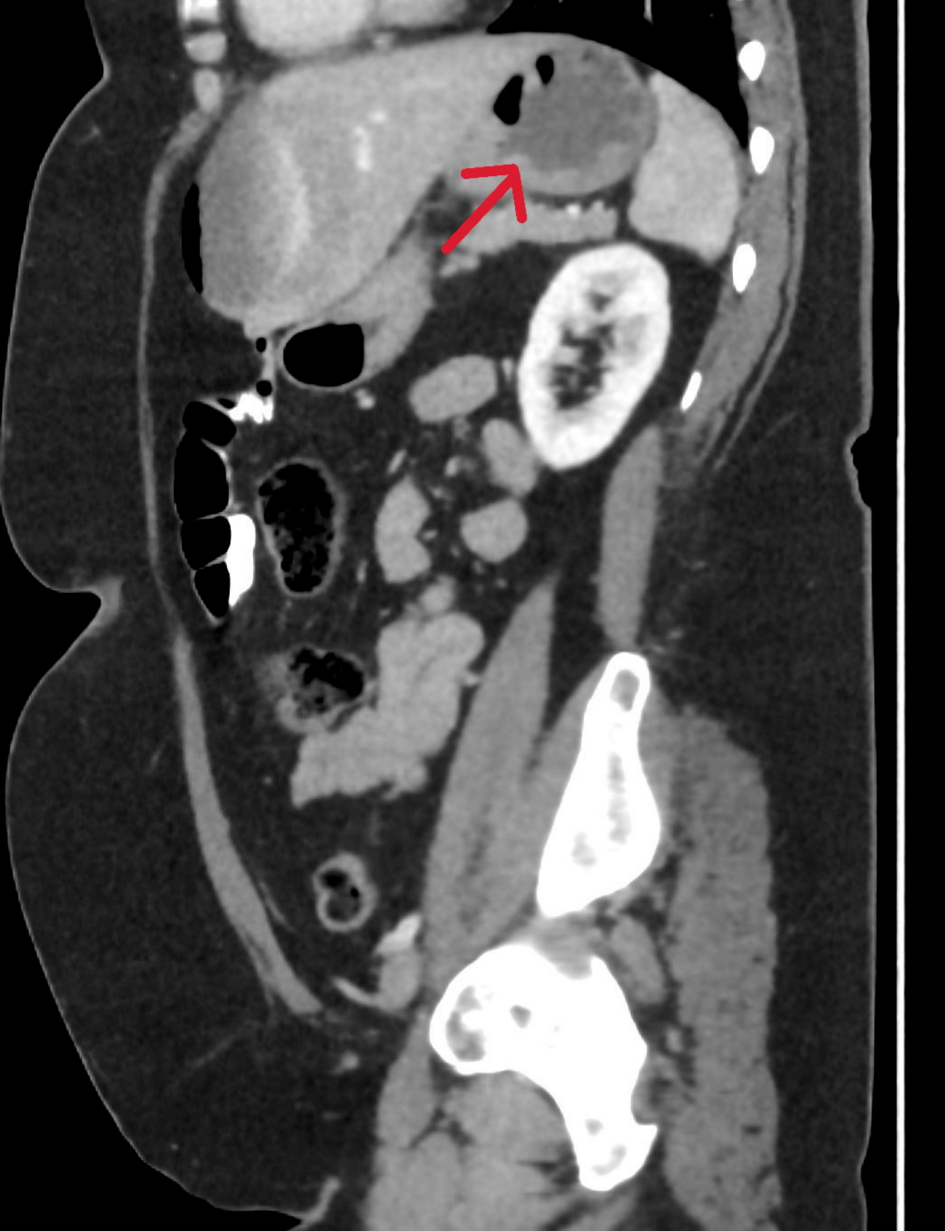
Sagittal CT scan revealing a well-defined perihepatic fluid collection consistent with a biloma

The patient was monitored for six hours post-procedure. During this period, she developed severe abdominal pain with generalized tenderness, requiring intravenous fentanyl. An urgent contrast-enhanced computed tomography (CT) scan of the abdomen revealed a newly developed epileptiform fluid collection with an air fluid level in the anterior left hepatic lobe, suggestive of biloma. Pneumobilia within the gallbladder was also noted, which is a commonly expected finding post-ERCP (Figures 2, 3).

She was commenced on intravenous piperacillin-tazobactam (4.5 g TDS) and metronidazole (500 mg TDS) along with intravenous fluids. After an anesthetic team review, a patient-controlled analgesia (PCA) was initiated.

The case was discussed with a tertiary centre, and it was decided for the patient to have IR drainage. Interventional radiology performed the insertion of a pigtail catheter into the subcapsular collection, aspirating ~250 ml of dark bile. Cytology showed blood and rare degenerated cells with the absence of malignant cells.

**Figure 4:**
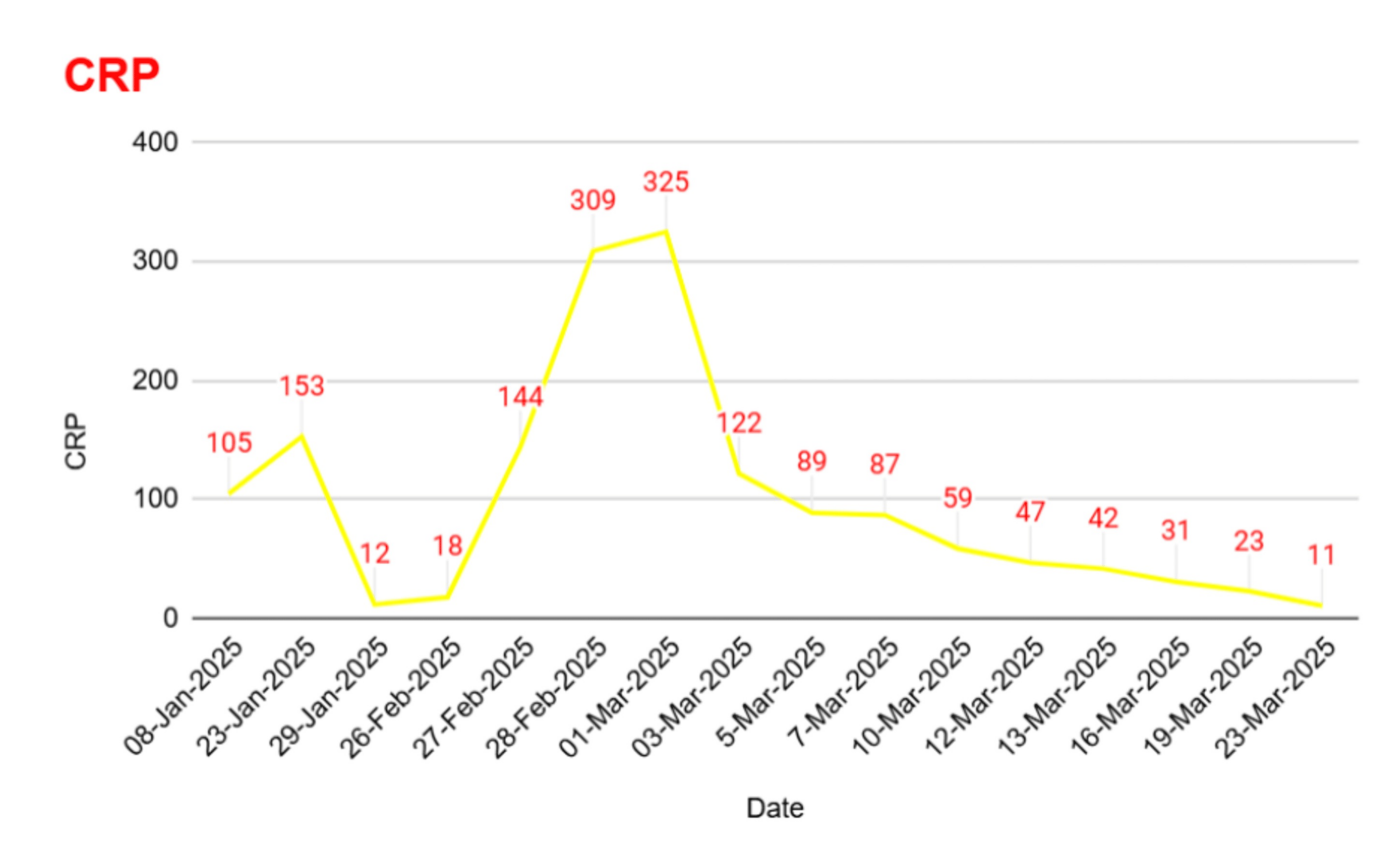
C-reactive protein (CRP) Trend

She subsequently developed hospital-acquired pneumonia (HAP) with right lower lobe consolidation, confirmed on chest X-ray. This was associated with desaturation and elevated inflammatory markers, for which Microbiology recommended continuation of current IV antibiotics (Figure 4).

**Figure 5:**
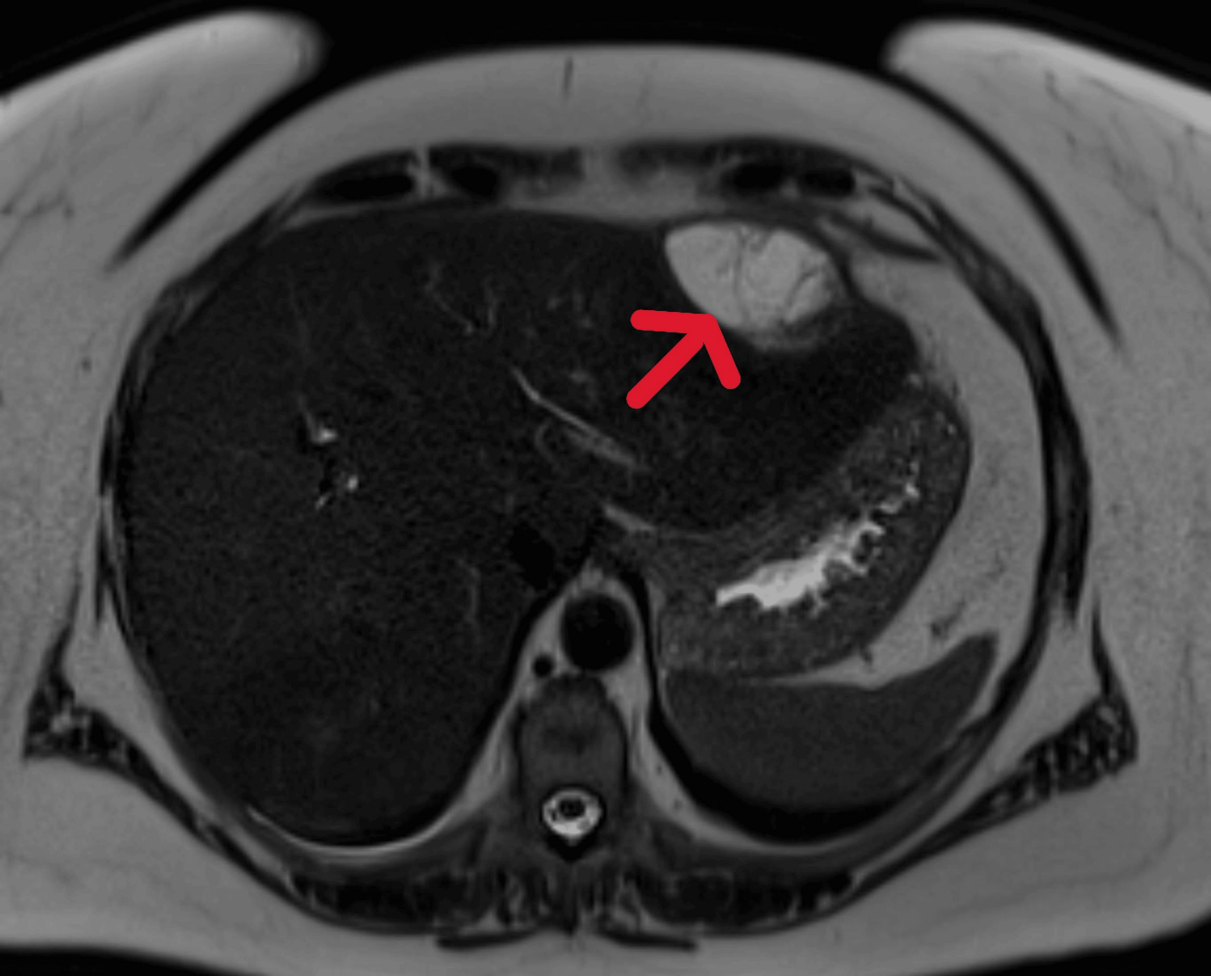
Magnetic resonance cholangiopancreatography (MRCP) showing a well-defined biloma

By day 14, antibiotics were stepped down to oral co-trimoxazole (960 mg BD) and metronidazole (500 mg TDS). A repeat MRCP on day 19 showed a persistent left subcapsular biloma with possible fistulous communication and a few residual CBD calculi (Figure 5). Specialist endoscopy advised repeat ERCP and continuation of drainage.

**Figure 6:**
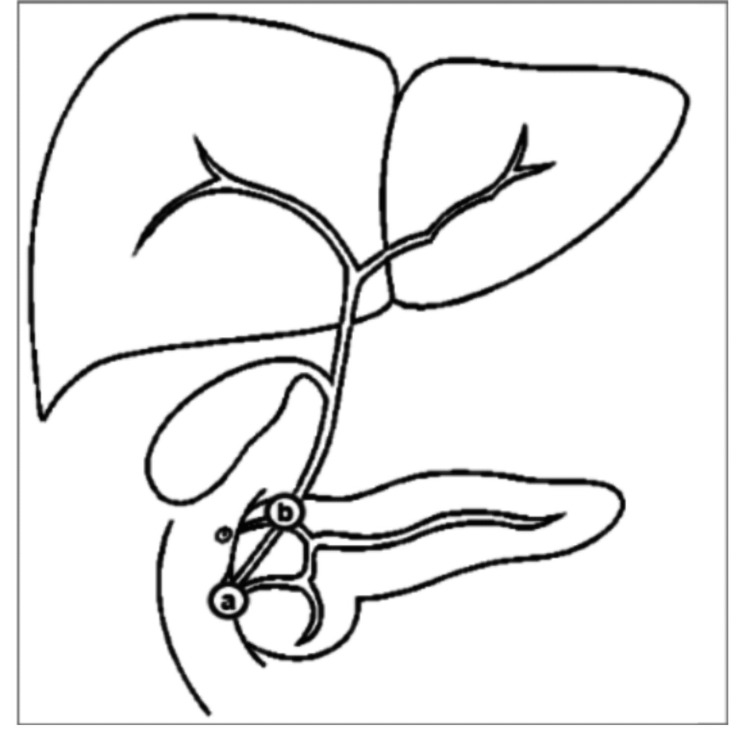
Figure showing the variant biliary anatomy (marked a to b) Image courtesy of Aintree University Hospital. Created for internal reporting purposes and reproduced here with permission.

Repeat ERCP on day 22 revealed two small CBD stones extracted via a balloon trawl. No extravasation or fistula was observed on occlusion cholangiogram. Notably, biliary anatomy was variant, with the right posterior duct draining directly into the cystic duct (Figure 6). Post procedure, oral antibiotics were continued, and drain output was monitored.

By day 26, the patient showed marked clinical improvement with downtrending ALP and inflammatory markers. The drain was removed after cessation of output, and she was discharged with a plan for elective cholecystectomy.

## Discussion

Bilomas, defined as localized collections of bile outside the biliary tree, are rare complications following ERCP, with subcapsular intrahepatic bilomas being particularly uncommon. ERCP is a common and effective way to treat stones in the common bile duct, and although complications like pancreatitis, bleeding, infection, and perforation are well recognized, injuries to the bile duct that lead to bilomas are rarely seen [1,2]. In our case, a previously healthy woman in her early 50s developed a left-sided subcapsular biloma within six hours post-ERCP, despite an otherwise uneventful procedure, highlighting how subtle or occult iatrogenic injury may lead to significant complications even in routine cases.

The mechanism of biloma formation in this context is believed to be due to micro-perforation or trauma to peripheral bile ducts from guidewire manipulation or balloon trawling, particularly when the biliary anatomy is variant [3]. In our patient, variant ductal anatomy was later confirmed, with the right posterior duct draining into the cystic duct, an anatomical variation known to increase procedural complexity and risk of ductal injury [4]. Notably, no extravasation was seen during the second ERCP, suggesting either spontaneous sealing of a microleak or extrabiliary accumulation via fistulous communication, as hinted by repeat MRCP findings.

The diagnosis of bilomas often relies on imaging, with contrast-enhanced CT being the modality of choice in acute settings, offering rapid identification of fluid collections and associated features such as pneumobilia [5]. In our case, CT performed within hours of symptom onset enabled early recognition of the biloma, facilitating prompt antibiotic therapy and interventional radiology-guided drainage. Subsequent infection with hospital-acquired pneumonia complicated the course, underlining the importance of vigilant inpatient monitoring post-ERCP, especially in patients requiring invasive drainage.

Management of post-ERCP bilomas is typically conservative or minimally invasive, involving broad-spectrum antibiotics and percutaneous drainage where necessary [6]. Our patient responded well to pigtail catheter insertion and tailored antibiotics, with no need for emergency surgical intervention. The second ERCP was critical in addressing residual CBD stones and confirming the absence of ongoing leak, allowing safe drain removal and discharge planning. This stepwise management approach, combining percutaneous drainage, broad-spectrum antibiotics, and follow-up ERCP, is supported by contemporary guidelines for post-ERCP bilomas. Current literature advocates for such minimally invasive strategies in hemodynamically stable patients without evidence of complex collections or systemic infection, with surgical intervention reserved for refractory cases [7,8]. In the present case, the second ERCP proved particularly valuable, both in addressing the underlying etiology (residual CBD stones) and confirming resolution of the biliary leak, which enabled successful drain removal.

This case emphasizes the need for awareness of rare complications after ERCP, especially with anatomical variations and multiple CBD stones. Early imaging and multidisciplinary coordination between gastroenterology, radiology, and infectious diseases teams played a key role in the patient's recovery. It also highlights how important it is to perform follow-up imaging and therapeutic ERCP in managing residual pathology and guiding clinical decisions.

## Conclusions

This case underscores the importance of suspecting subcapsular biloma as a rare but serious post-ERCP complication. With early recognition using imaging, a multidisciplinary team approach including interventional radiology, proper antimicrobial therapy, and repeat ERCP led to a faster patient recovery. This also emphasizes the need to be familiar with biliary anatomical variants, as this is crucial for anticipating and managing unexpected complications during ERCP. 
